# Breathing-Adapted Imaging Techniques for Rapid 4-Dimensional Lung Tomosynthesis

**DOI:** 10.1016/j.adro.2023.101173

**Published:** 2023-01-18

**Authors:** Arielle A. Uejo, Michael G. Snyder, Joseph T. Rakowski

**Affiliations:** a Department of Oncology, Karmanos Cancer Institute, Flint, MI; b Department of Radiation Oncology, Beaumont Hospital, Royal Oak, MI; c Department of Oncology, Karmanos Cancer Institute, Wayne State University School of Medicine, Detroit, MI

## Abstract

**Purpose:**

This article presents enhancements to a 4-dimensional (4D) lung digital tomosynthesis (DTS) model introduced in a 2018 paper. That model was proposed as an adjunct to 4D computed tomography (4DCT) to improve tumor localization through artifact reduction achieved by imaging the entire lung in all projections, reducing the projection collection time duration for each phase compared with 4DCT, and requiring only a single-breath cycle to capture all phases. This is applicable to SABR treatment planning. Enhancements comprise customized patient 4D-DTS x-ray scanning parameters.

**Methods and Materials:**

Imaging parameters derived with the 4D-DTS model were arc duration, frames per second, pulse duration, and tube current normalized to single-chest radiographic milliampere-seconds (mA/mAs_AEC_). Optimized phase-specific DTS projections imaging parameters were derived for volunteer respiration-tracking surrogate waveforms and for sinusoidal waveforms. These parameters are temporally matched to the respiratory surrogate waveform and presented as continuous data plots during a period of 20 seconds. Comparison is made between surrogate excursions during a single-phase CT and 4D-DTS reconstructions.

**Results:**

4D-DTS imaging techniques were customized to volunteer respiratory waveforms and sinusoidal waveforms. Technique settings at the highest velocity portions of the volunteer waveforms were arc duration 0.066 seconds, frame rate 921 Hz, pulse duration 1.076 ms, and normalized tube current 76.2 s^–1^. Technique settings at the highest velocity portions of the sinusoidal waveforms were arc duration 0.029 seconds, frame rate 2074 Hz, pulse duration 0.472 ms, and normalized tube current 173.6 s^–1^. Sinusoidal surrogate excursion distance at the highest velocity portion of the waveform during a CT rotation of 0.5 seconds ranged from 2.68 to 21.09 mm, all greater than the limiting excursion distance chosen in the 4D-DTS model.

**Conclusions:**

4D-DTS image technique settings can be customized to individual patient breathing patterns so that captured range of motion satisfies an operator-selected value.

## Introduction

An earlier paper presented a chest 4-dimensional (4D) digital tomosynthesis (DTS) model operating at high projection acquisition rates that would allow capture of lung tissue motion at any phase with user-selected maximum tissue excursions, with fewer motion artifacts than 4D computed tomography (4DCT), emphasizing application to stereotactic body radiation therapy (SBRT) treatment planning but assisting and not replacing 4DCT.^1^ Other investigators have studied the potential use of tomosynthesis in radiation therapy but limited it to 30 frames per second.^2^, ^3^, ^4^, ^5^, ^6^, ^7^, ^8^, ^9^

Chest tomosynthesis is also being investigated for use in interventional electromagnetic navigation bronchoscopy (ENB) guidance and transbronchial biopsy via endobronchial ultrasonography with a guide sheath with studies showing that chest tomosynthesis could mitigate the divergence between the ENB and planning CT acquired days or weeks before the intervention.^10^, ^11^, ^12^, ^13^, ^14^, ^15^, ^16^, ^17^ Chest tomosynthesis is also being used to evaluate the severity of respiratory stenosis, to confirm positioning after respiratory stenting, and to confirm the position of the endobronchial Watanabe spigot during bronchial occlusion.^17^ 4D-DTS may eliminate the ENB limitation of the need for a breath-hold maneuver or neuromuscular blockade when using fluoroscopic-based c-arm tomosynthesis to allow imaging sans motion.^18^

The intent of the original model was to complement radiation therapy 4DCT by reducing lesion localization uncertainty in sagittal and coronal planes. This is achieved by imaging the entire lung in all projections, reducing the projection collection time duration for each phase relative to 4DCT, and taking advantage of finer longitudinal spatial resolution of the flat panel imager relative to 4DCT. Clinical implementation would require sharing of the Digital Imaging and Communications in Medicine (DICOM) space between the 4D-DTS device and CT to facilitate locating the appropriate 4D-DTS reconstruction planes, possibly by longitudinally sharing the patient couch between the devices.

The original model relied on innovations of cold cathode electron emitters with pulse durations of approximately 0.16 to 2.0 ms, flat panel detector variable frame rates of approximately 500 to 6000 s^–1^, and ideal sinusoidal breathing waveforms. This report refines the model by determining patient-specific x-ray pulse duration, frame rate, arc duration, and tube current from volunteer breathing waveforms and simulated sinusoidal waveforms.

Sources of 4DCT image artifacts can be divided into the categories of irregular breathing cycles and inherent spatial and temporal limitations of 4DCT technology. Additionally, Watkins et al found that artifacts due to intraphase residual motion exist in 4DCT even for ideal breathing motions, determined that those artifacts depend on patient-specific tumor motion and CT gantry rotation speed, and developed an ideal periodic motion-based cine-mode 4DCT motion-based model of geometric uncertainty due to partial projection artifacts.^19^ In other words, the object being imaged moves as the CT gantry rotates at a fixed speed, limiting object localization due to gantry rotation time and velocity of the object. Their model also recommends adding an additional inherent uncertainty of one slice thickness. Clements et al found that large and varied motion displacements can reduce 4DCT and cone beam CT (CBCT) internal target volumes (ITVs) and lengths: a lesion moving irregularly and reaching its maximum displacement when outside the 4DCT imaging plane will not be visible in the reconstruction, and a lesion traveling to a large displacement once during cone beam CT imaging will be averaged out of the final reconstruction.^20^ Yamamoto et al studied a population of 50 radiation therapy patients and found high frequency and magnitudes of multislice cine 4DCT artifacts categorized as blurring, duplicate, overlapping, and incomplete.^21^

## Methods and Materials

### Breathing waveform acquisition

Respiration waveforms of graduate student volunteers were acquired using the Varian RPM system (Varian Medical Systems, Palo Alto, CA) without x-ray exposure. Simulated sinusoidal waveforms were also generated for comparison.

### Projection imaging

The original model proposed 61 projections through a linear arc angle ≥40 degrees delivered by a linear array of individual focal spots. Radiographic autometic exposure control (AEC) for an average-size chest anterior-posterior image was used to set baseline $mAs_{AEC}.$

Milliampere-seconds (mAs) per tomographic projection was computed as

(1) $$mAs\;per\;projection=\frac{\left(mAs_{AEC}\right)\;\times \;f}{NP\;per\;sweep\;per\;energy}$$

where NP is the number of projections per arc and f = 5 or 2.5 is a noise scaling factor with 10 regarded as clinically acceptable in chest DTS.^22^ Values less than 10 are used in the model to reduce tube current demands, which will also reduce patient dose and signal to noise ratio. The value 10 was chosen here because it is the manufacturer's recommendation for the GE VolumeRAD chest tomosynthesis system (GE Healthcare, Chicago, IL) that has been studied in several publications. For example, Hwang et al conducted a 4-observer phantom study of variation in patient dose and lung nodule detection with the GE Volume RAD tomosynthesis unit using 10:1 and 5:1 dose ratios (referred to as noise scaling factor in this paper), both at combinations of 3 kVp values and 2 filtrations.^23^ They found a 56.7% dose reduction with 5:1 versus 10:1 dose ratio and no significant change in detection of 4 mm or larger nodules.

Zhang et al studied dose between chest radiography, tomosynthesis, and CT for adult patients. In their paper, they referenced the 10:1 dose ratio from the GE VolumeRAD unit user manual.^24^ Sharma et al computed tomosynthesis organ dosimetry dose coefficients for the GE VolumeRAD.^25^ Bath et al created a method to estimate patient tomosynthesis dose-area product for the GE VolumeRAD.^26^

### 4D-DTS model respiration waveform analysis

The original model proposed estimating optimal pulse duration, pulse rate, frame rate, and tube current using the adjustable but idealized waveform model of Lujan et al, but only at the maximum surrogate velocity.^27^ In this report, imaging parameter waveforms are derived from volunteers’ breathing waveforms measured with the Varian RPM system (Varian Medical Systems), as well as simulated breathing waveforms. Imaging parameters are temporally matched to the respiratory surrogate waveform and presented as continuous data plots during a period of 20 seconds.

A DTS projection frame rate (s^–1^) adequate for limiting craniocaudal motion to a predetermined distance during capture of an individual phase depends on the following: (1) breathing surrogate displacement b; (2) the desired maximum surrogate motion capture during the arc D; (3) number of projections per arc NP; and (4) phase-dependent tissue velocity, quantified here by surrogate motion. In clinical application, surrogate motion must be transformed to diaphragm motion for proper application of the model.

Image quality depends, among other things, on the number of projections, arc angle, mAs per projection, and kilovoltage.^28^

Arc duration, frame rate, x-ray pulse duration, and tube current derived from actual patient data would be based on the average breath tracking surrogate velocity over intervals of 0.5 seconds in the breathing waveform. The measured velocity concept here assumes the surrogate waveform velocity equals the longitudinal velocity of tissue nearest the diaphragm. However, surrogate motion should be correlated with true diaphragm motion before applying this model.^29^, ^30^, ^31^, ^32^, ^33^, ^34^

The average velocity is determined as

(2) $$V_{average}=\frac{1}{N}\sum_{i=\;1}^{N}\left[\frac{A\left(t_{i}\right)\;-\;A\left(t_{i-1}\right)}{t_{i\;}-\;t_{i-1}}\right]$$

where $A(t_{i})$ is the breathing displacement at time $t_{i},$ with *N* = 15, waveform sampling time interval $t_{i}-t_{i-1}$ of 0.033 seconds, and averaging interval of 0.495 seconds.

The computed model imaging techniques results are presented in this paper to coincide with a maximum tissue travel *D =* 1 mm during a single arc, and with the noise scaling factor f = 5 and baseline chest radiographic mAs value $mAs_{AEC}.$ Corrections based on these parameters can be applied to the present data to derive techniques for different values of f, D, and $mAs_{AEC}.$

Arc duration for a maximum diaphragm travel at time *t* (seconds) is determined as

(3) $$Arc\;duration=\;D\times {\left(V_{average}\left(t\right)\right)}^{-1}\;\left(\text{seconds}\right)$$

Limits were placed on this arc duration so that there is no overlap in time from one sampled breathing phase or displacement to the next sampling point in time. Overlaps can occur when *V_average_(t)* is small. These limits were chosen to fit the breathing surrogate waveform, and manifest as flat portions of the arc duration, frame rate, pulse duration, and tube current plots presented in the Results section.

The frame rate (Hz) at time *t* was computed as

(4) Framerate=NP/(Arcduration)(Hz)

where NP is the number of projections per arc.

(5) $$Pulse\;duration=\left[\left(\frac{1}{Frame\;rate}\right)-Frame\;gap\right]$$

where frame gap is time duration between consecutive projections:

(6) $$Frame\;gap=\left(\frac{1}{100,000\;fps}\right)=\;0.01\;ms$$

Normalized pulse tube current per $mAs_{AEC}$ is

(7) $$mA=\frac{mAs\;per\;projection}{\left(Pulse\;duration\right)\;\times \;\left(mAs_{AEC}\right)}$$

### Intraphase motion analysis

A comparison will be made between surrogate excursions during a single-phase CT reconstruction and 4D-DTS reconstruction. Correlation between actual diaphragm motion and the surrogate waveform is not made here but would be necessary for model implementation.

### Volunteers

Surrogate maximum velocity and displacement ΔS of volunteer surrogate breathing waveforms were evaluated using Equation 2, with displacement (ie, amount of blur), determined as

(8) $$\Delta S\left(t\right)=V{\left(t_{max}\right)}_{average}\times \delta$$

where $t_{max}$ is the time value at which average velocity is maximum and δ is gantry rotation time of 0.5 and 0.28 seconds.

### Simulations

Simulated breathing waveforms were generated using the Lujan model^27^:

(9) $$z\left(t\right)=z_{0}-b\times cos^{2n}\left(\pi t\;/\;\tau -\emptyset_{0}\right)$$

where

z(t) = SI position at time t;

$z_{0}$ = position at exhale, assigned value 0;

b = displacement of motion;

$z_{0}-b$ = position at inhale;

τ = period of breathing cycle;

n = parameter that determines the waveform steepness and flatness, assigned value 1; and

$\emptyset_{0}$ = starting phase of the breathing cycle, assigned value 0.

The instantaneous velocity at time t is

(10) $$\frac{dz\left(t\right)}{dt}=\frac{2n\pi b}{\tau }cos^{2n-1}\left(\pi t\;/\;\tau \right)sin\left(\pi t\;/\;\tau \right)$$

Simulated maximum tissue velocities and motion ranges during gantry rotation were investigated by 2 methods: (1) by the method of Equation 8 and (2) by analytically taking the difference in Equation 9 displacement values over the period δ centered around the time value at which instantaneous velocity is maximum.

## Results

### Derived projection techniques

**Figure 1:**
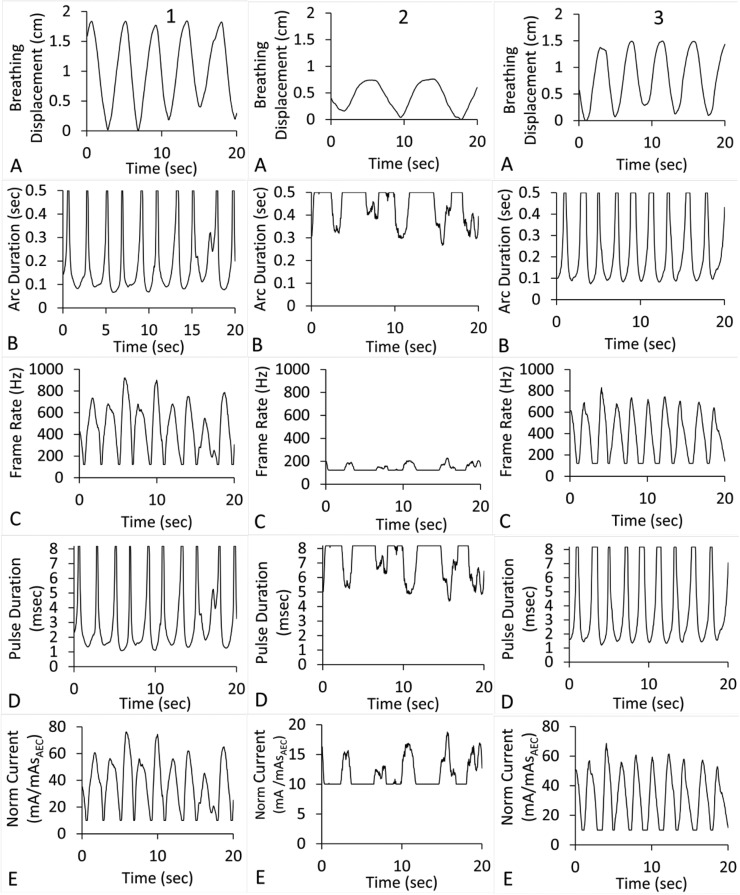
Volunteers’ surrogate breathing waveforms 1 through 3 and associated imaging technique plots for *D* = 1 mm and *f* = 5. (A) Breathing displacement (cm) versus time. (B) Arc duration (seconds). (C) Frame rate (Hz). (D) Pulse duration (ms). (E) Normalized tube current (mA/mAs_AEC_).

Figure 1 presents volunteers’ respiration waveforms along with the associated plots of arc duration, frame rate, pulse duration, and tube current to mAs_AEC_ ratio $\left(mA/mAs_{AEC}\right)$ for conditions D = 1 mm, NP = 61, f = 5. This technique data can be extended to other conditions by using appropriate ratios of old and new values of f, D, NP, and $mAs_{AEC}.$ These image acquisition technique plots were computed per Equations 3 through 7 using the average velocity (Equation 2) of the breathing displacement waveforms over intervals of 0.495 seconds. Using intervals less than 0.495 seconds to compute velocity leads to an unreasonable number of high gradient fluctuations in the derived technique plots over a single-breath cycle especially for an irregular surrogate breathing waveform. X-ray pulse and projection arc maximum durations were restricted to prevent overlaps in time and allow temporal gaps between frames. These manifest as flat regions of the technique plots.

**Figure 2:**
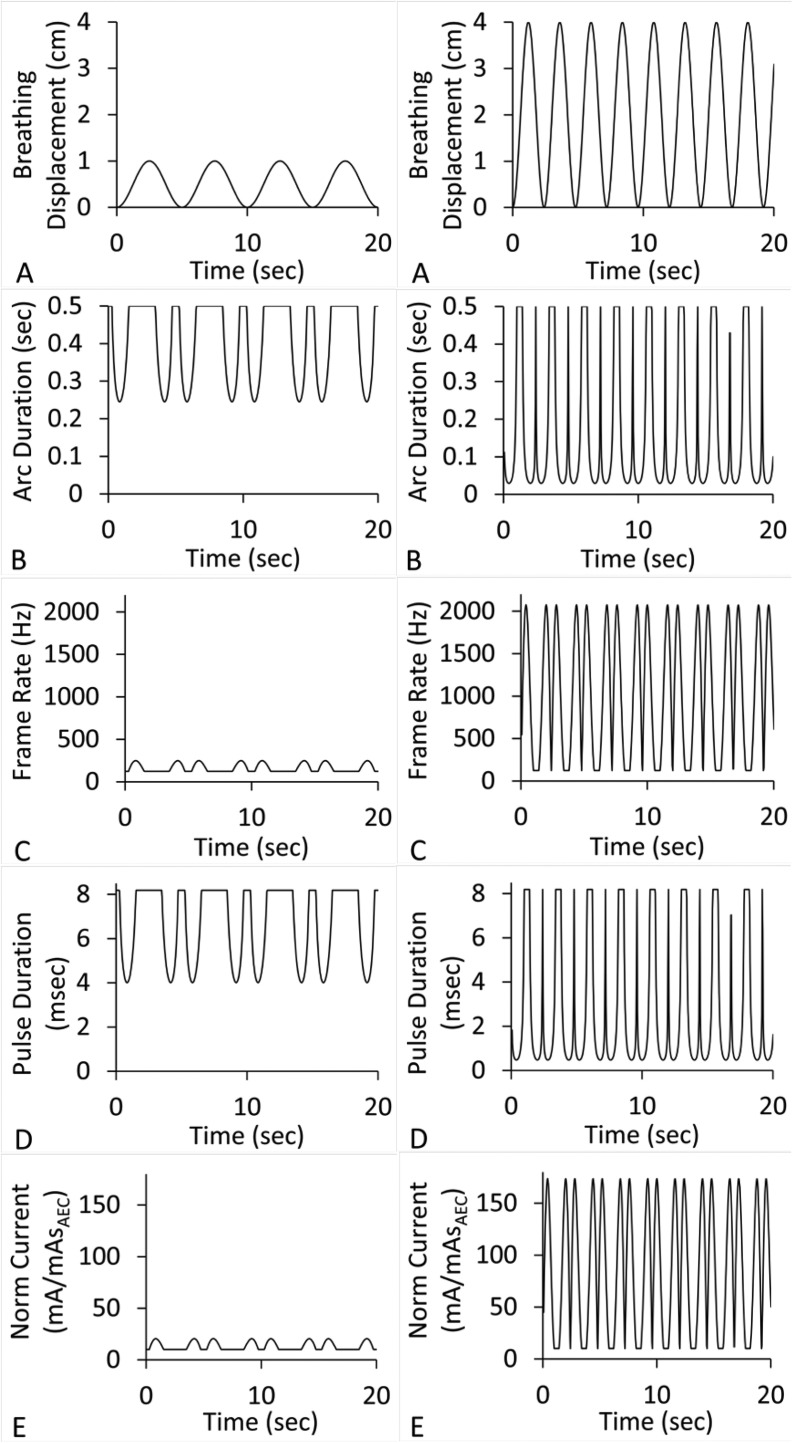
Simulated sinusoidal breathing waveforms and associated imaging techniques plots for *D* = 1 mm and *f* = 5. Left: 1 cm displacement, 12 bpm. Right: 4 cm displacement, 25 bpm. (A) Breathing displacement (cm) versus time. (B) Arc duration (seconds). (C) Frame rate (Hz). (D) Pulse duration (ms). (E) Normalized tube current (mA/mAs_AEC_). *Abbreviation:* bpm = breaths per minute.

Figure 2 presents sinusoidal waveforms derived from the sinusoidal-based model of Lyman et al and the associated technique waveforms. Velocity was computed by Equation 10 for breathing patterns of 10 mm displacement, 12 breaths per minute (bpm), and 40 mm displacement, 25 bpm. In these sinusoidal patterns the model parameter that determines general steepness and flatness n was set equal to 1, which generates a sine function. An n > 1 value creates greater steepness and flattens the exhale portion. Increased steepness would increase the demands on the DTS model imaging technique values.

**Figure 3:**
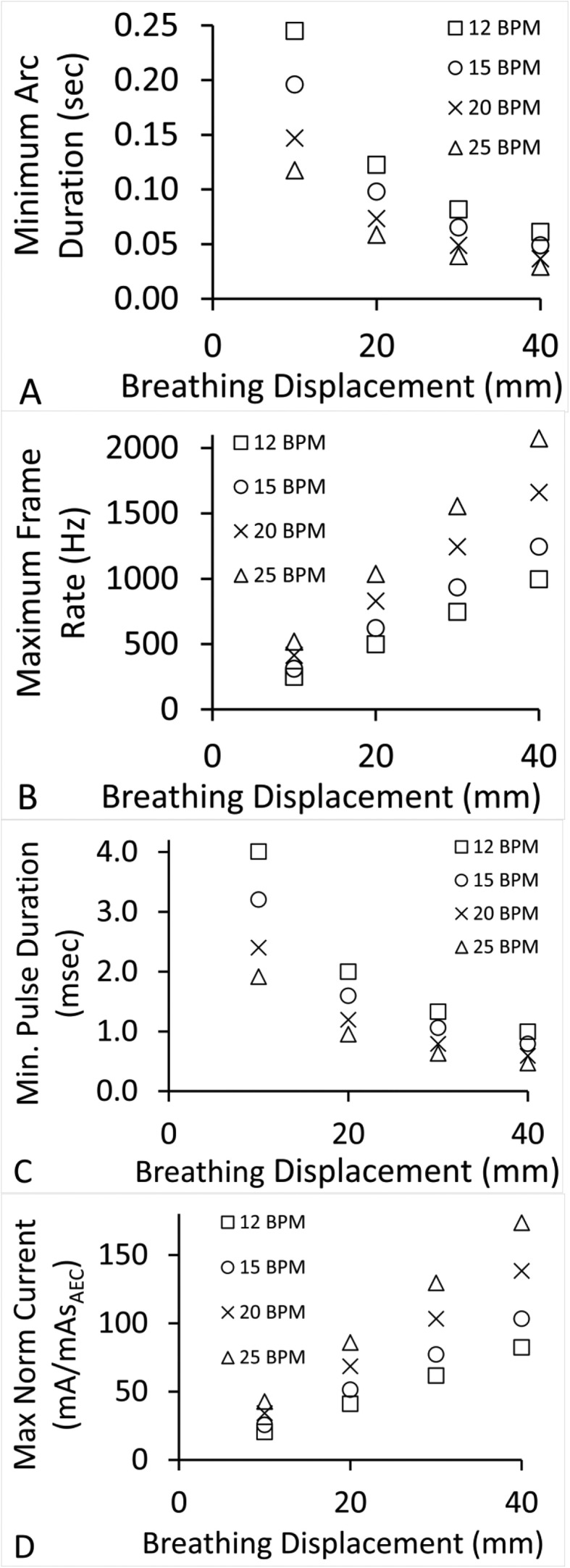
Imaging technique extreme values derived from simulated sinusoidal breathing waveforms using instantaneous analytical velocity at 12, 15, 20, and 25 breaths per minute and 10 to 40 mm breathing displacements for *D* = 1 mm and *f* = 5. (A) Minimum arc duration (seconds). (B) Maximum frame rate (Hz). (C) Minimum pulse duration (ms). (D) Maximum normalized tube current (mA/mAs_AEC_).

Figure 3 presents plots of extreme imaging technique values derived from the sinusoidal-based model of Lujan et al with velocity computed as it was for Fig. 2 with breathing displacements of 10, 20, 30, and 40 mm and respiration rates of 12, 15, 20, and 25 bpm.^27^

Table 1 presents the extreme scanning parameters for *D* = 1 and f = 5 for volunteers and simulated breathing cycles: (1) volunteers’ extreme values of shortest arc duration of 0.066 to 0.267 seconds, fastest frame rate of 228 to 921 Hz, shortest x-ray pulse duration of 1.076 to 4.368 ms, and maximum tube current, normalized to $mAs_{AEC}.$ of 18.8 to 76.2 s^–1^; (2) simulated sinusoidal waveforms scanning parameters, using the average velocity (Equation 2), of shortest arc duration 0.034 to 0.253 seconds, fastest frame rate 241 to 1801 Hz, shortest x-ray pulse duration 0.545 to 4.141 ms, and maximum tube current, normalized to $mAs_{AEC}.$ 10.0 to 150.3 s^–1^; (3) simulated sinusoidal waveforms scanning parameters, using the instantaneous velocity (Equation 10), of shortest arc duration 0.029 to 0.245 seconds, fastest frame rate 249 to 2074 Hz, shortest x-ray pulse duration 0.472 to 4.007 ms, and maximum tube current, normalized to $mAs_{AEC}.$ 20.4 to 173.6 s^–1^.

**Table 1:** Extreme scanning parameter values, *D* = 1 mm and *f* = 5

Volunteers’ maximum surrogate linear model average velocity				
	Arc duration (s)	Frame rate (Hz)	Pulse duration (ms)	Normalized tube current mA/mAs_AEC_ (s^–1^)
1	0.066	921	1.076	76.2
2	0.267	228	4.368	18.8
3	0.073	830	1.195	68.6

Simulated maximum surrogate intraphase residual linear model motion average velocities				
Waveform displacement and rate	Arc duration (s)	Frame rate (Hz)	Pulse duration (ms)	Normalized tube current mA/mAs_AEC_ (s^–1^)

1 cm, 12 bpm	0.253	241	4.141	10.0
1 cm, 25 bpm	0.135	450	2.211	37.1
4 cm, 12 bpm	0.063	964	1.028	79.7
4 cm, 25 bpm	0.034	1801	0.545	150.3

Simulated maximum surrogate intraphase residual motion analytical instantaneous velocities				
Waveform displacement and rate	Arc duration (s)	Frame rate (Hz)	Pulse duration (ms)	Normalized tube current mA/mAs_AEC_ (s^–1^)

1 cm, 12 bpm	0.245	249	4.007	20.4
1 cm, 25 bpm	0.118	519	1.918	42.7
4 cm, 12 bpm	0.061	995.8	0.994	82.4
4 cm, 25 bpm	0.029	2074	0.472	173.6

*Abbreviation:* bpm = breaths per minute.

Table 2 presents the maximum surrogate intraphase motion velocities and ranges. Maximum surrogate intraphase motion distances for maximum velocity breathing were the following: (1) volunteers at maximum average velocity, 1.87 to 7.55 mm for the 0.5-second 360-degree CT rotation period and 1.05 to 4.23 mm for the 0.28-second period; (2) simulated sinusoid at maximum average velocity, 1.97 mm at 1 cm excursion, 12 bpm to 14.76 mm at 4 cm, 25 bpm for the 0.5-second period and 1.11 mm at 1 cm excursion, 12 bpm to 8.27 mm at 4 cm, 25 bpm for the 0.28-second period; (3) simulated sinusoid at maximum instantaneous velocity, 2.68 mm at 1 cm excursion, 12 bpm to 21.09 mm at 4 cm, 25 bpm for the 0.5-second period and 1.52 mm at 1 cm excursion, 12 bpm to 12.41 mm at 4 cm, 25 bpm for the 0.28-second period.

**Table 2:** Maximum surrogate intraphase motion velocities and ranges

Volunteers’ intraphase linear model motion			
Volunteer waveform no.	Maximum average velocity (mm/s)	0.5 s range (mm)	0.28 s range (mm)
1	15.10	7.55	4.23
2	3.74	1.87	1.05
3	13.61	6.81	3.81

Simulated intraphase linear model motion (Lujan model)			

Simulation Parameter	Maximum average velocity (mm/s)	0.5 s range (mm)	0.28 s range (mm)

1 cm, 12 bpm	3.949	1.97	1.11
1 cm, 25 bpm	7.380	3.69	2.07
4 cm, 12 bpm	15.798	7.90	4.42
4 cm, 25 bpm	29.522	14.76	8.27

Simulated intraphase linear model motion (Lujan model)			

Simulationparameter	Maximum instantaneous velocity (mm/s)	0.5 s range (mm)	0.28 s range (mm)

1 cm, 12 bpm	4.081	2.68	1.52
1 cm, 25 bpm	8.502	5.27	3.10
4 cm, 12 bpm	16.324	10.71	6.07
4 cm, 25 bpm	34.009	21.09	12.41

*Abbreviation:* bpm = breaths per minute.

## Discussion

Irregular breathing creates artifacts in 4DCT. Regular motion in a perfect respiratory waveform creates blur in the CT phase image reconstruction because projections must be collected during the gantry rotation period, either a full 360-degree scan as was assumed here, or a partial scan of less than 360 degrees that can however have associated artifacts. As described by Watkins et al, the slice thickness is an added uncertainty to these distances.^19^ In comparison, 4D-DTS limits the maximum surrogate intraphase residual motion distance per phase to a user-prescribed value, here assigned 1 mm, with an additional image plane uncertainty determined by pixel pitch which is consistently less than 4DCT reconstructed slice thickness.

Imaging the entire lung in every projection, requiring no more than a single-breath cycle to capture all phases, will remove motion-related artifacts, provided the single-breath cycle is continuous. Several cycles of varying displacements and rates can be recorded before imaging to improve the predictive algorithm learning and performance. Single-breath cycles can be imaged at varying displacements to capture lung motion variation.

The derived arc duration, frame rate, pulse duration, and tube current can be extracted from any respiration waveform using the method in Equations 2 through 7. These can be applied in different ways. For example, these imaging parameters may be actively adjusted during imaging by a predictive algorithm trained by the patient's prerecorded waveform.^35^, ^36^, ^37^, ^38^, ^39^, ^40^, ^41^, ^42^, ^43^, ^44^, ^45^, ^46^ Alternatively a single set of extreme values of each parameter derived from the patient's extended waveform could be used at each phase throughout the projection acquisitions (ie, the shortest arc duration, fastest frame rate, shortest x-ray pulse duration, and maximum tube current).

Data in Figs. 1 to 3 and Table 1 demonstrate that greater maximum displacement and bpm values will increase demands on the 4D-DTS imaging system. Table 2 demonstrates that greater maximum displacement and bpm values also affect uncertainty in tissue position in reconstructed phases, with additional uncertainty contributed by reconstruction slice thickness. In comparison, maximum 4D-DTS reconstruction spread due to motion is predetermined at the time of imaging, assumed to be ≤1 mm in this study, with additional spread provided by the pixel pitch typically <0.3 mm.

Use of 4D-DTS in conjunction with 4DCT can aid in more precise segmentation of lung lesion if the DTS system shares the DICOM space with the CT scanner, which will allow exact spatial correlation between CT and DTS reconstructions, probably by longitudinally transferring the patient between devices on the fixed CT table in the same suite. The depth of the 4D-DTS reconstruction slice can be selected on the axial 4DCT reconstructions per phase, with finer spacing of the 4D-DTS reconstruction planes in a slab centered on the selected target. Deformation of the 4DCT reconstructed transverse view to match the 4D-DTS surrogate respiratory motion signal may be possible before 4D-DTS target selection to improve correlation between 4DCT and 4D-DTS. This process would require correlation of internal respiratory motion with surrogate signals.^29^, ^30^, ^31^, ^32^, ^33^

Inherent spatial uncertainty in 4D-DTS is dependent primarily on the user's choice of excursion capture distance and motion during imaging. The excursion distance in this model, being based on the surrogate waveform, should be converted to the actual diaphragm motion, which in turn will be equal to or greater than lung tissue excursion.^29^, ^30^, ^31^, ^32^, ^33^ Diaphragm motion can be correlated to the waveform by imaging a limited area at the diaphragm using a single focal spot of the 4D-DTS system. A future challenge is contending with the slight breathing motion present in the 4D-DTS projections during reconstructions. The limited depth resolution in DTS could be overcome by simultaneously imaging in the orthogonal plane.^1^^,^^7^

A further enhancement to this model could be dual-energy DTS which would demand a higher frame rate in the image receptor, increased electron current (mA) at the x-ray source, shorter pulse length, and a technique to rapidly change the kV whether by doubling the number of focal spots or rapidly changing the accelerating voltage. Gomi et al have studied dual energy tomosynthesis of the lung.^47^, ^48^, ^49^ Radiomics combined with uninterrupted free-breathing motion information of the full lung provided by rapid 4D-DTS may prove useful in diagnosing diseases affecting lung elasticity.

## Conclusion

To summarize, rapid 4D-DTS image technique settings can be customized to individual patients’ breathing patterns. Longitudinal position uncertainty due to artifacts common in 4DCT can be reduced using 4D-DTS image information.
